# Analysis of thermochemical energy storage in an elemental configuration

**DOI:** 10.1038/s41598-019-52249-8

**Published:** 2019-11-04

**Authors:** Alexandre Malley-Ernewein, Sylvie Lorente

**Affiliations:** 1 LMDC, Université de Toulouse, UPS, INSA, Toulouse, France; 2 Villanova University, Department of Mechanical Engineering, Villanova, PA 19085 USA

**Keywords:** Energy storage, Energy science and technology, Engineering

## Abstract

Here we show theoretically that the design of a thermochemical energy storage system for fast response and high thermal power can be predicted in accord with the constructal law of design. In this fundamental configuration, the walls of the elemental cylinder are impregnated with salt, while humid air is blown through the tube. Cases with constant salt volume or constant fluid volume or both are considered. It is shown that the best design in each case meets the equipartition of imperfections principle. The predictions are confirmed by full numerical experiments, allowing to consider various shape ratios and study their impact on the overall performance.

## Introduction

In 2015, the Paris climate summit led to the agreement that clean energy and decarbonized energy systems were a necessity. The building sector is under focus: in 2017, 27% of the final energy consumption in Europe was spent in households while 64.1% of the household consumption was due to space heating. Among those 27%, the renewables represent 18%^1^. Increasing the share of renewables requires to work on several paths: more technological and scientific advances are essential, the entire society must be conscious of the need and accept to be part of the change, political measures must sustain the trend… Doing better means also a continuous decrease in the cost of renewable energy while solving the mismatch between energy production and consumption. It is a well-known challenge: increasing the share of the renewables in the energy mix leads to increasing the burden of bridging the gap between production and consumption periods, a gap with several time scales: a peak demand of a few hours during day time, usually corresponding to the most expensive moment of electricity production, and heating needs of several weeks during the cold season (if not the entire winter season). This in turn poses the issue of the storage volume which may reach values unacceptable by the users. In sum the question is not about harnessing the energy but rather how to do it in an efficient fashion when space becomes premium.

Among the available energy storage technologies, Thermochemical Energy Storage appears promising, allowing (i) higher energy densities compared to sensible or phase change materials storage, and (ii) no heat leakage. A careful screening was made in N’Tsoukpoe *et al*.^2^ among 125 salts, based on several criteria including toxicity. Strontium Bromide (SrBr2) appeared to be one of the top candidate. The main disadvantage appears when rather thick salt layers are tested (several cms) as the hydration of salt grains leads to porosity changes at the expenses of the process thermal efficiency. We are interested in open systems where humid air is used as the heat transfer fluid. Note that depending on the case the inlet fluid temperature may be obtained through the combination with solar thermal energy harvesting^3^. Therefore, the solid-gas thermochemical reaction can be summarized as “Salt + Water vapor $\rightleftharpoons$ Hydrated salt + reaction enthalpy”. If research intensified in the last decade^2,4–16^, we observe that it was through the design of prototypes mostly based on trial and error, and that the fundamental question on how to distribute the available space between the reacting salt and the heat transfer fluid for energy efficiency was not addressed yet.

The observation of the natural evolution of flow systems towards configurations offering easier access to their currents was stated for the first time in 1996 through the constructal law^17^. In constructal design, the flow must be understood in its broadest assertion: a flow happens every time a potential difference is created. It may be a flow of fluid, of heat, of mass etc. The morphing of flow architectures for least overall flow resistance was then proven to be predictable. Examples are found in all the domains of science: from engineering and the tree-shaped configurations of point-to-volume flow systems^18^, to biology and the prediction of animal life span^19,20^, medicine^21^ and most recently the quantum footprint^22^.

In this paper we use the constructal law to demonstrate that the design of thermochemical energy storage for energy efficiency can be predicted. We focus on a small elemental component to shed light on the fundamental aspects of transport phenomena: a cylindrical channel which wall is impregnated with a reactive salt. This elemental system is aimed at being a component of a bigger system honeycombe shaped.

## Results

**Figure 1:**
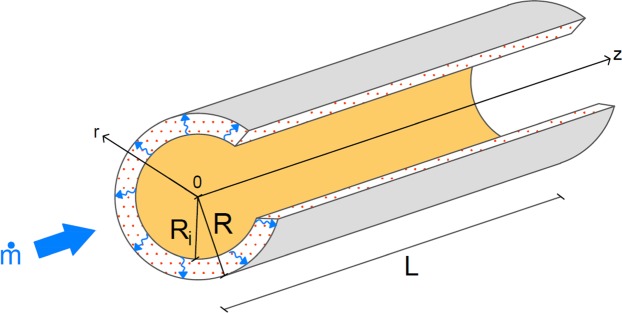
Elemental configuration: the heat transfer fluid is channeled through the tube letting water vapor diffuse radially through the salt impregnated along the tube wall.

Here we consider a cylinder of radius *R* and length *L*. A volume of salt *V*_*s*_ is deposited uniformly along the cylinder wall. We hypothesise that one layer of salt grain covers the wall and will consider only diffusion through the grain. Assume that the salt impregnation on the internal tube wall is done at grain scale. The heat transfer fluid (humid air) licks the salt, water vapor diffuses within, allowing the chemical reaction to happen, and heat to be stored or released. The salt thickness is termed *e*. The volume *V*_*f*_ of humid air blown along the cylinder is *V*_*f*_ = *πR*_*i*_^2^*L*. We have *R*_*i*_ + *e* = *R* (Fig. 1).

**Figure 2:**
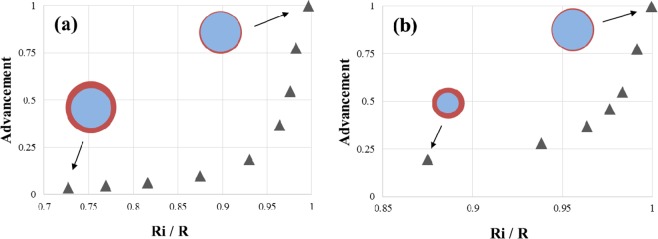
The reaction advancement at *t* = *t*_(*a*=0)_ + Δ*t*, when (**a**) the fluid volume is fixed, and (**b**) the salt volume is fixed.

The inlet boundary conditions ($\dot{m}$,*T*_*in*_,*c*_*in*_) remains constant. The salt thickness is chosen so that it corresponds to the order of magnitude given in Eq. (5), meaning that the time for diffusion through the salt matches the time for the chemical reaction to happen. Figure 2 shows the value of the advancement for *t* = *t*_(*a*=0)_ + Δ*t* in two cases. The advancement represents the ratio between the mass of hydrated salt at time t and the mass of fully hydrated salt. At first, the fluid volume was fixed, together with the channel length (Fig. 2a). The thickness of impregnated salt varies such that the radii ratio covers the range 0.727 < *R*_*i*_/*R* $\lesssim$ 1. Decreasing *R*_*i*_/*R* means increasing the salt thickness. Next, the channel length and the salt volume remained constant. The tube diameter through which humid air flows is then a degree of freedom (Fig. 2b). The range of diameters ratio explored is 0.875 < *R*_*i*_/*R* $\lesssim$ 1. Note that the increase in the reaction advancement becomes steeper when *R*_*i*_/*R* → 1 in all the cases.

**Figure 3:**
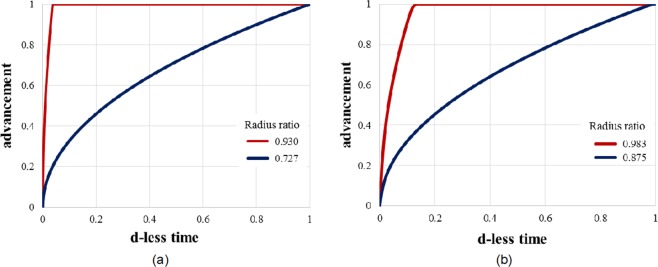
The reaction advancement as a function of the dimensionless time for 2 extreme cases when (**a**) the fluid volume is fixed, and (**b**) the salt volume is fixed.

**Figure 4:**
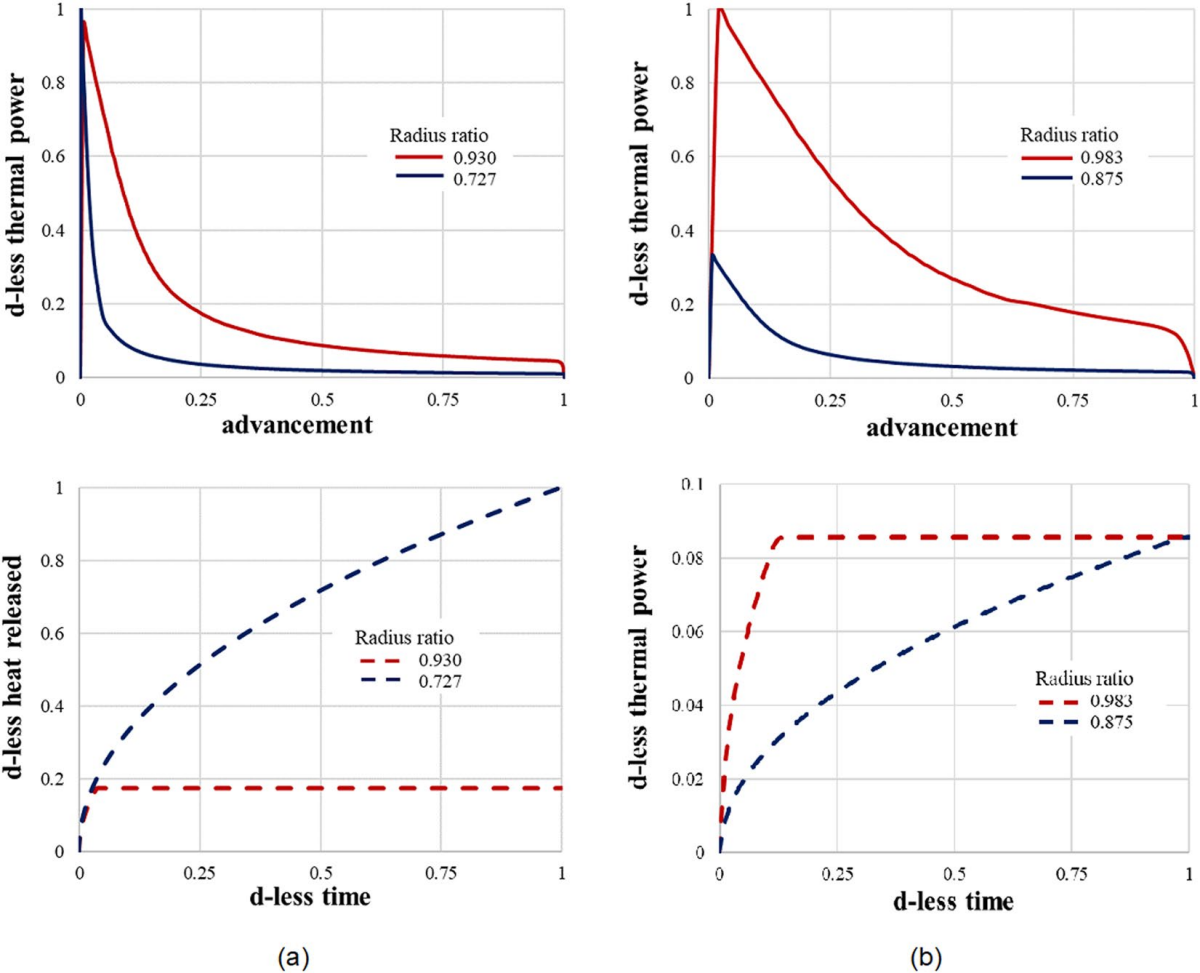
The dimensionless thermal power as a function of the advancement (top), and the dimensionless heat released as a function of the dimensionless time (bottom) for 2 extreme cases when (**a**) the fluid volume is fixed, and (**b**) the salt volume is fixed.

To get more insight on the effect of *R*_*i*_/*R*, Fig. 3 shows the reaction advancement as a function of time for some of the extreme cases. They are *R*_*i*_/*R* = 0.727, and *R*_*i*_/*R* = 0.930 when the fluid volume is fixed (Fig. 3a), and *R*_*i*_/*R* = 0.875, and *R*_*i*_/*R* = 0.983 when the salt volume is fixed (Fig. 3b). In both cases, the reaction advancement increases with the radii ratio: the thinner the total salt thickness, the higher the advancement of the chemical reaction. The thermal power produced by the salt reaction is shown in a non-dimensional way in Fig. 4a,b (top) as a function of the reaction advancement. The d-less power is calculated by dividing the thermal power by the maximum value obtained in each configuration (fixed fluid volume or fixed salt volume), which corresponds to the case with the smallest radius ratio. The corresponding energy is also determined ($\Delta h{n}_{s}{V}_{s}a(t)$). It varies linearly with the reaction advancement, and the salt volume. More information on the kinetics of heat released appears when plotting the results as a function of the d-less time (see Fig. 4a,b, bottom).

More challenging is the case where the salt volume is fixed together with the fluid volume. Note that the fluid volume is linked to the salt volume through *V*_*s*_ = *π*(*R*_*i*_ + *e*)^2^*L*−*V*_*f*_. The channel inner radius and the salt thickness are estimated from the theoretical analysis based on the constructal law (see Methods). As the volumes are prescribed, the channel length is not a degree of freedom anymore. Several configurations are picked: one corresponding to the recommendations obtained from the constructal law in terms of salt thickness (Eq. (5)) and radius ratio, see Eq. (7). The other configurations continue to obey the radius ratio order of magnitude, yet, the salt thickness is either smaller or bigger that the one dictated by Eq. (5). Consequently, the channel length will be respectively longer or smaller.

**Figure 5:**
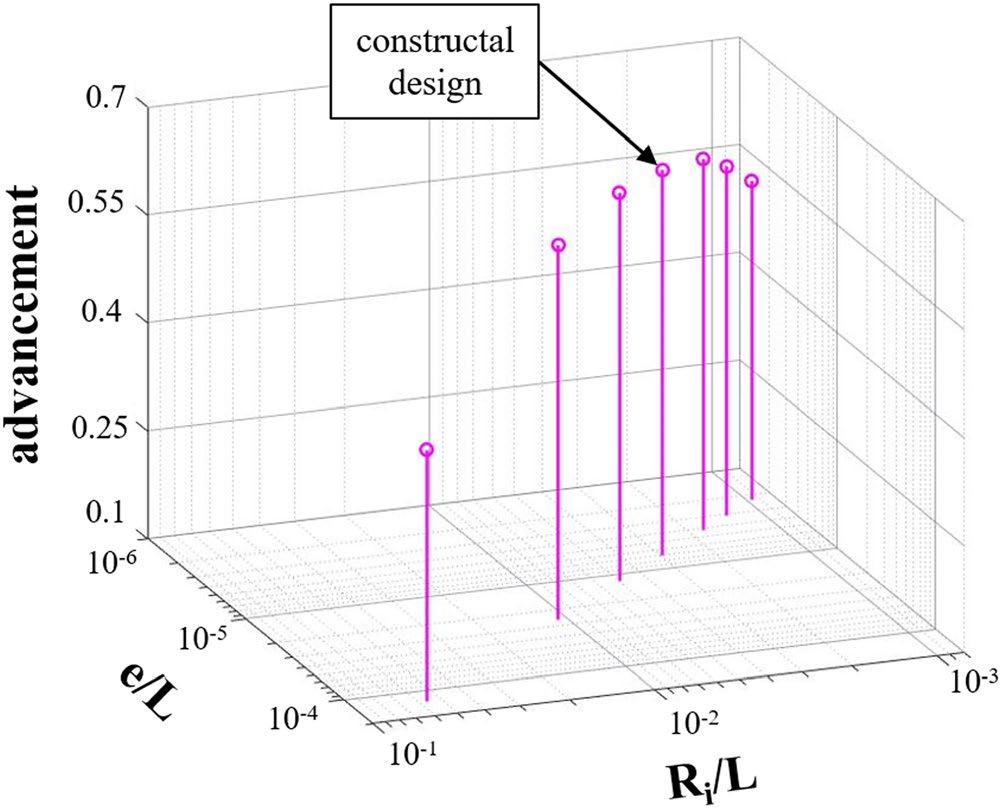
The reaction advancement as a function of the shape ratios e/L and R_i_/L with fixed salt and fluid volumes at t/t_(a=1)_ = 0.141.

**Figure 6:**
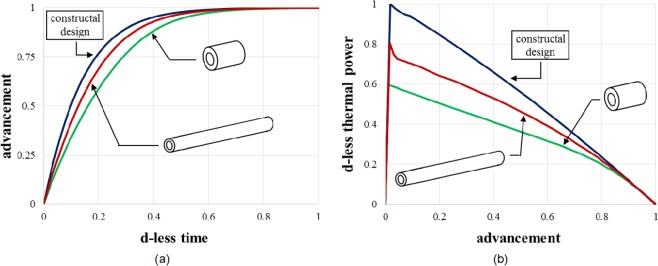
The comparison between constructal design and 2 extreme cases (short and large, long and slender): (**a**) reaction advancement as a function of the dimensionless time, and (**b**) thermal power as a function of the advancement.

We see in Fig. 5 the evolution of the advancement for a d-less time (t/t_(a=1)_ = 0.141), and various ratios *R*_*i*_/*L* and *e*/*L*, while the salt and fluid volumes are always maintained constant. The time was chosen only for the sake of illustration. The trend is identical for other values of time. This figure shows the impact of the geometry on the reaction advancement, and therefore the system performance. Plotted also is the configuration corresponding to the constructal arrangement. The following figure (Fig. 6a,b) focuses on the two extreme cases (*R*_*i*_/*L* ≅ 5 · 10^−2^ with e/L ≅ 1.3 · 10^−4^, and *R*_*i*_/*L* ≅ 10^−3^ with e/L ≅ 2.3 · 10^−6^) surrounding the theoretical results obtained from the constructal approach, and presents the results in terms of advancement and thermal power. The constructal design is always superior.

## Discussion

Thermochemical Energy Storage finds utility in different building applications. The way to design the reactor must be adapted to the objective and the time-scale of the storage. When the fluid volume is fixed, or when the salt volume is fixed, a higher channel radius ratio leads to a faster chemical reaction. The constructal law predicts that *R*_*i*_/*R* should be close to 1, see Eq. (7), provided the salt thickness is in the order of magnitude given by Eq. (5). For a fixed fluid volume a thinner layer of impregnated salt allows a much faster reaction (Fig. 3a) as the time for diffusion through the salt is proportional to the square of the salt thickness. Yet the peak value of the released thermal power is quasi identical, regardless the radius ratio, thus the salt volume.

When the salt volume is fixed (with constant tube length) we see in Fig. 3b how the advancement changes in time for 2 extreme cases. An increase in the channel radius corresponds to a decrease in the salt thickness which comes closer to value predicted in Eq. (5), while the exchange surface at the interface between the salt and humid air increases. The impact of the salt arrangement on the released thermal power is noticeable in Fig. 4b where the power is plotted as a function of the advancement.

The heat extracted is directly proportional to the salt volume. This is why it is much higher when *R*_*i*_/*R* = 0.727 than *R*_*i*_/*R* = 0.930 (for constant fluid volume), Fig. 4a. For a constant salt volume (Fig. 4b) the heat released is the same whatever the salt geometrical configuration. Important is the way heat leaves the channel in time: fast for a high radius ratio *R*_*i*_/*R*, in a much smoother manner for a smaller *R*_*i*_/*R*.

If the objective is to produce heat as fast as possible in order to contribute to shaving the heating peak demand, the results indicate that a large volume of salt may not be necessary (for constant fluid volume), as a small salt volume allows the thermochemical reaction in the fastest way possible, provided the salt thickness obeys Eq. (5). On another hand, increasing the volume of impregnated salt becomes a solution for providing heating during a prescribed period of time.

Moving to constant fluid volume and constant salt volume requires to work on the radii aspect ratio but also on the radius/length aspect ratio. The design that meets the recommendations dictated by the constructal law allows a faster reaction advancement (Fig. 5). Decreasing the salt thickness comes with increasing the channel length. As the fluid residence time increases accordingly, the reaction of the entire salt volume is slower. Shortening the tube length means to increase the salt thickness; this creates an imbalance between the time for molecular diffusion through the salt and the time for the chemical reaction to happen (Fig. 6a). Morphing the system in accord with the trends predicted by the theoretical approach leads to more thermal power released even though the salt volume available for the thermochemical reaction is constant. Note in Fig. 6b that its peak value is significantly higher.

## Methods

### Constructal configuration

Mass transfer throughout the salt is mainly radial as the salt thickness (grain scale) is much smaller than the fluid radius. Therefore mass conservation gives^23,24^

1 $$\frac{\partial c}{\partial t}\cong {D}_{v,s}\frac{{\partial }^{2}c}{\partial {r}^{2}}+\gamma \frac{\partial {c}_{s}}{\partial t}$$

where *c* is the water vapor concentration, *D*_*v*,*s*_ is the vapor diffusion coefficient through the salt, and *γ* is a stoichiometric coefficient.

Considering Fick’s diffusion alone through the salt of thickness *e*, the scale analysis of Eq. (1) gives

2 $${t}_{diff} \sim \frac{{e}^{2}}{{D}_{v,s}}$$

where *t*_*diff*_ is the time scale of diffusion. The reaction time of the salt (*t*_*reaction*_) represents the time necessary for the the chemical reaction to happen. It is given, in an order of magnitude sense, by the time when the water vapor concentration decreases due to the chemical reaction.

3 $$\frac{\Delta c}{{t}_{reaction}} \sim \gamma {n}_{s}{k}_{scin}$$

where *n*_*s*_ is the salt molar density and *k*_*cin*_ is the kinetic constant.

The constructal law states that the optional allocation of the material volume obeys an equipartition of time principle^25,26^, which means here that the time for vapor diffusion through the salt grains *t*_*diff*_ must match the reaction time of the salt volume *t*_*reaction*_. Hence, if in an order of magnitude sense, $\Delta c \sim {n}_{s}$

4 $$e \sim {(\frac{{D}_{v,s}}{\gamma {k}_{cin}})}^{1/2}$$

If not,

5 $$e \sim {(\frac{{D}_{v,s}}{\gamma {k}_{cin}}\frac{\Delta c}{{n}_{s}})}^{1/2}$$

Equations (4) and (5) show that the salt thickness should be chosen as a function of the expected vapor concentration drop between inlet and outlet (in an hydration configuration), $e \sim \sqrt{\Delta sc}$, which is a measure of the number of vapor moles reacting with the salt.

The heat gained by the fluid is given by ${m}_{f}{c}_{p}\Delta T$ The heat released during the hydration of the salt is $\Delta h\,{n}_{s}{V}_{s}$ where $\Delta h$ is the reaction enthalpy.

Energy conservation imposes

6 $${\rho }_{f}{V}_{f}{c}_{p}\Delta T \sim \Delta h\,{n}_{s}{V}_{s}$$

And after some math,

7 $${(\frac{R}{{R}_{i}})}^{2}-1 \sim \frac{{\rho }_{f}{c}_{p}\Delta T}{\Delta h\,{n}_{s}}$$

or

8 $${(1+\frac{e}{{R}_{i}})}^{2} \sim 1+\frac{{\rho }_{f}{c}_{p}}{\Delta h\,{n}_{s}}\Delta T$$

Equation (8) unravels the relationship that exists between *e*/*R*_*i*_, the ratio of salt thickness and channel radius, and the change in fluid temperature.

More information on the channel geometry can be obtained by considering that the heat transfer rate along the channel is $\dot{m}{c}_{p}\Delta T$, with $\dot{m} \sim \frac{{\rho }_{f}{V}_{f}}{{t}_{f}}$ and *t*_*f*_ the fluid residence time in the duct. Maximum heat transfer density happens when the fluid residence time matches the time for thermal diffusion in the channel

9 $${t}_{f} \sim \frac{{{R}_{i}}^{2}}{{\alpha }_{f}}$$

The salt hydration heat rate is $\Delta h\frac{\partial {c}_{s}}{\partial t}{V}_{s}$, where $\frac{\partial {c}_{s}}{\partial t}={n}_{s}\frac{\partial a}{\partial t}$ and $\frac{\partial a}{\partial t}$ is the advancement kinetic of the chemical reaction. We have^27^

10 $$\frac{\partial a}{\partial t}={k}_{cin}(1-\frac{{p}_{eq}}{{p}_{v}})(1-a)$$

where *p*_*v*_ is the vapor pressure, and *p*_*eq*_ is the equilibrium gas pressure. The latter is a function of the temperature through the Clausius-Clapeyron equation^28^.

From scale analysis, we write $\frac{\partial a}{\partial t} \sim {k}_{cin}$. As *V*_*f*_ ~ *LR*_*i*_^2^, the first law of thermodynamics writes

11 $${k}_{f}\,L\Delta T \sim {k}_{cin}\Delta h\,{n}_{s}{V}_{s}$$

and

12 $${R}^{2}-{R}_{i}^{2} \sim \frac{{k}_{f}\Delta T}{\,{k}_{cin}\Delta h\,{n}_{s}}$$

Combining Eqs (7) and (12) together, we obtain

13 $${{R}_{i}}^{2} \sim \frac{{\alpha }_{f}}{{k}_{cin}}$$

and

14 $${R}^{2} \sim \frac{{\alpha }_{f}}{{k}_{cin}}(1+\frac{{\rho }_{f}{c}_{p}\Delta T}{{n}_{s}\Delta h})$$

Finally, making use of Eqs (5) and (13)

15 $${(\frac{e}{{R}_{i}})}^{2} \sim \frac{{D}_{v,s}\Delta c}{\gamma \,{n}_{s}{\alpha }_{f}}$$

### Model

In order to test the theoretical results obtained from constructal design, a numerical model was developed on a finite elements platform^29^. The heat transfer fluid was humid air, and the salt was SrBr_2_. The main physical characteristics are provided in Table 1.

**Table 1:** Fluid and Solid characteristics.

Humid air	density^29^, kg/m^3^	*ρ*_*f*_	1.179
	dynamic viscosity^29^, Pa.s	*μ*	1.830 · 10^−5^
	heat capacity^29^, J/(kg.K)	*c*_*pf*_	1011.9
	thermal conductivity^29^, W/(m.K)	*k*_*f*_	0.026
Water vapor	diffusion coefficient in air^33^, m^2^/s	*D*_*v*, *f*_	2.6 · 10^−5^
	diffusion coefficient through the salt grains^34^, m^2^/s	*D*_*v*, *s*_	1.2 · 10^−13^
Salt	density^30^, kg/m^3^	*ρ*_*s*_	*ρ*_0_ + *a*(*ρ*_1_−*ρ*_0_)*ρ*_0_ = 3481*ρ*_1_ = 2390
	heat capacity^30^, J/(kg.K) (subscript 0 and 1 stand respectively for fully dehydrated and fully hydrated)	*c*_*ps*_	${c}_{{p}_{s,0}}+a({c}_{p,{s}_{1}}-{c}_{p,{s}_{0}})$ ${c}_{p,{s}_{0}}=456$ ${c}_{p,{s}_{1}}=968$
	molar density^30^, mol/m^3^ (subscript 0 and 1 stand respectively for fully dehydrated and fully hydrated)	*n*_*s*_	4144.8
	thermal conductivity^30^, W/(m.K)	*k*_*s*_	1

The model equations are given by the following conservation laws:

(i) Mass conservation of humid air in the channel:
  16 $$\frac{D{\rho }_{f}}{Dt}=0$$
  where *ρ*_*f*_ is the humid air density. The changes in water vapor content do not impact the humid air properties^30^.
(ii) Mass conservation of water vapor in the channel (necessary to link with the water vapor diffusion through the salt):
  17 $$\frac{Dc}{Dt}+\nabla (-{D}_{v,f}\nabla c)=0$$
  where c is the water vapor concentration, and *D*_*v*,*f*_ is the vapor diffusion coefficient in the fluid (air).
(iii) Mass conservation of water vapor in the salt:
  18 $$\frac{\partial c}{\partial t}+\nabla (-{D}_{v,s}\nabla c)=-\,\gamma \,{n}_{s}\frac{da}{dt}$$
(iv) Momentum conservation of humid air in the channel, in laminar regime:
  19 $$\frac{D{\boldsymbol{u}}}{Dt}=-\frac{1}{{\rho }_{f}}\nabla p+\nu {\nabla }^{2}{\boldsymbol{u}}$$
  where ***u*** is the velocity vector.
(v) Energy conservation along the tube:
  20 $${\rho }_{f}{c}_{pf}\,\frac{DT}{Dt}+\nabla \cdot ({k}_{f}\,\nabla T)=0$$
(xvi) Energy conservation through the salt:
  21 $${c}_{ps}\frac{\partial T}{\partial t}+\nabla \cdot ({k}_{s}\nabla T)={n}_{s}\frac{da}{dt}\varDelta h$$
(xvii) Reaction kinetic:

22 $$\frac{da}{dt}={k}_{cin}(1-\frac{{p}_{eq}}{{p}_{v}})(1-a)$$

The temperature *T*_*in*_ and the water vapor concentration *c*_*in*_ were fixed at the entrance of the fluid cylinder. To assume a fully developed flow at the inlet, an entrance length greater than 0.05 *Re*(2*R*_*i*_) was imposed^31^, for an inlet mass flow rate $\dot{m}$. The outlet was at atmospheric pressure, and an outflow condition was applied as a boundary condition for heat transfer. The walls of the cylinder (*r* = *R*_*i*_ + *e*) were thermally insulated without any vapor flux. A no-slip condition was imposed at the interface between the fluid and the salt (*r* = *R*_*i*_).

At t = 0, the initial temperature of the system was *T*_*init*_, the fluid was at rest and at atmospheric pressure. The initial vapor concentration was *c*_*init*_ corresponding to the equilibrium vapor pressure at temperature *T*_*init*_.

Due to the axial symmetry, the three-dimensional geometry was simplified to a 2D axi-symmetry one. The model meshing was optimized in a dedicated study where the energy and mass conversation were used as verification criteria. The model was validated by comparing its results to an experimental study of an open system in which parallelepiped layers of SrBr_2_ were submitted to 7 cycles of hydration and dehydration. The input data were well controlled: moist air temperature, vapor pressure and volumetric air flow rate. More details can be found in Malley-Ernewein and Lorente^32^.
